# Caught Between Now and Next: A Qualitative Study into Final-Year Medical Students’ Clerkship Choices

**DOI:** 10.5334/pme.1747

**Published:** 2025-11-14

**Authors:** Alexandra Androni, Jeanine G. Bosma, Bettine T. ter Haar, Marco A. de Carvalho Filho, Esther Meijer

**Affiliations:** 1 Wenckebach Institute for Education and Training (WIOO), LEARN – Lifelong Education and Assessment Research Network, University Medical Centre Groningen, University of Groningen, The Netherlands; 2 Department of Health Psychology, University Medical Center Groningen, University of Groningen, Groningen, The Netherlands; 3 Wenckebach Institute for Education and Training (WIOO), Faculty of Medical Sciences, University Medical Centre Groningen, University of Groningen, The Netherlands; 4 Department of Nephrology, University Medical Center Groningen, University of Groningen, Groningen, The Netherlands

## Abstract

**Introduction:**

Choosing a final clerkship that suits their goals and aspirations is felt as one of the most important decisions by medical students. Understanding preferences for medical students’ clerkship choices and the factors that drive them may help clinical educators ensure optimal support throughout students’ clinical training. Our objective was to identify what factors influence medical students’ clerkship choices.

**Methods:**

We conducted individual semi-structured interviews with 16 final-year medical students enrolled in clinical clerkships. We analyzed the data under a constructivist paradigm, using template analysis, a form of thematic analysis.

**Results:**

Students highlighted four main themes influencing the choice of their final clerkship: “emotions involved in making the decision”, “between now and next”, “the learning environment beyond the clerkship” and “decisions under structural pressures”. A climate of high competition, peer pressure and haste seem to add to the difficulty of choosing a clerkship.

**Discussion:**

When choosing their clinical clerkships, final year medical students prioritize immediate well-being, social support, and a sense of belonging, searching for ‘a good work-life balance’ and ‘an informal collegial work environment’.

## Introduction

Medical education requires students to engage in a wide range of clinical experiences during their training. Clinical clerkships provide medical students with early exposure to real-world clinical scenarios they are likely to encounter after graduation. This hands-on experience marks a critical shift from the classroom teaching to the clinical environment [1]. During this stage, students are expected to establish learning goals, which include performing clinical procedures on real patients and refining their bedside manner [2].

Significantly, medical students assume the role of clerks during clinical practice, allowing them to observe physician-patient interactions, practice clinical skills, and engage in decision-making. This practical involvement not only boosts their confidence but also helps them feel integrated into the medical profession [3–4]. As medical students progress through their clinical clerkships, they make multiple choices, one of the main professional ones being the deliberate selection of their final-year clinical placement(s) [5].

As such, clerkship rotations are fundamental for clinical training. The clinical learning environment is a complex domain that combines education with patient care, which naturally involves uncertainties [6]. Conducting a clerkship involves not only acquiring medical knowledge and skills but also engaging with fellow students, healthcare professionals, and patients. These clinical experiences play a crucial role in shaping students’ sense of professional and personal self [7–8]. Personal values also influence the development of future physicians, especially through interactions with family, friends, clinicians and other healthcare staff they meet during their clerkships [9–10].

Understanding how students make choices about elective rotations provides insight into what they expect from and value in their clerkship placement(s). Voluntarily choosing a clerkship often involves students evaluating a range of medical specialties they are exposed to, and then narrowing down the fields they find appealing [11]. This often leads to a process of elimination, where students reject clinical fields that do not align with their interests or abilities, rather than positively selecting specialties that offer an ideal fit [12]. Individual competencies [13], intellectually stimulating content [14], and professional aspirations [15–16] have all been cited as most directive towards medical students’ placement choices. After all, literature has shown that early placement preferences, particularly during clerkships, can be significant predictors of students’ eventual careers [17–18].

In addition to the above, it is important to acknowledge that students pay close attention to the informal work cultures and clinical team dynamics they are part of [19]. In the verge of internalizing the role associated with the medical profession, students critically align their personal values with their colleagues’ expectations [20]. Multiple authors support that relationships between students and residents or attending physicians play a pivotal role in validating students’ self-perceptions of competence [21], exposing them to elements of the hidden curriculum [22], and offering insights into the realities of different specialties [23]. Such elements are vital to students’ development, especially during the exploratory phase of clinical clerkships, when career intentions remain highly flexible.

To explore how medical students construct their career trajectories, we ought to deepen our understanding of their preferences and differences as they develop clinical pedagogy. By understanding what students seek, expect and grapple with in their clinical placements, clinical educators and curriculum designers can more effectively mentor students in navigating these critical and impactful decisions. This study therefore sought to answer the following research question: What factors influence final-year medical students’ decision-making in choosing their clinical clerkships?

## Methods

### Study design

We conducted an exploratory qualitative study, adopting a constructivist paradigm [24] which acknowledges reality as an active construction emerging from the interaction of the experiences of both participants and researchers. We used template analysis as our guiding framework [25], which directed the gathering and examination of a dataset of individual interviews with final-year medical students. As a flexible form of thematic analysis, template analysis combines a structured hierarchical coding process with the ability to respond to the nuances of a particular dataset. It begins with the development of an initial coding template, typically based on a subset of the data, which is then iteratively refined as analysis progresses. This study was carried out in accordance with the Declaration of Helsinki. Ethical approval was obtained from the ethical review board of the Netherlands Association of Medical Education (NVMO), dossier number: 2024.1.1.

### Study context

We conducted this interview study at the University of Groningen (UG), University Medical Center Groningen (UMCG), the Netherlands. The 6-year undergraduate medical curriculum of the University of Groningen is facilitated in Dutch and comprises a 3-year Bachelor’s (pre-clinical) phase and a 3-year Master’s (clinical) phase. Students are assessed on and expected to demonstrate Dutch language proficiency. The Bachelor’s program is problem-based and student-centred, with a focus on seminar groups, practicals and lectures. After completing the bachelor’s phase, students undertake a clinical placement program comprising an additional 3 academic years (M1, M2, M3). During the first two years of the clinical training (M1, M2), students are randomly assigned to various departments and hospitals by the faculty coordinators. These clerkships can be intramural or extramural, with a duration of four weeks each. Intramural clerkships take place within a hospital setting, whereas extramural clerkships are based in out-of-hospital healthcare facilities (i.e., GP practices, rehabilitation centers). Once in M3 (year 6 of the medical curriculum), students have the option to choose their own clinical placement, a module worth 28 European Credit Transfer System (ECTS) credits, divided into a core elective clerkship of 14 weeks and a short elective clerkship of 6 weeks. The choices of both clerkships are assessed by the faculty’s final clerkship committee and are thereupon approved or disapproved. The other half of the sixth year is reserved for the master’s thesis or dissertation, and the order in which students complete their final clerkship and thesis is flexible, allowing them to tailor their trajectory according to their preferences and circumstances.

### Participants

Participants were sixth-year medical students enrolled at the UG/UMCG who had their final clerkship request recently (<12 months) approved. Potential participants received an email invitation from the secretary of the final clerkship committee asking if they were willing to participate in the study. We employed the snowball technique, asking participants who first responded to our email to encourage colleagues from different healthcare settings (e.g., hospitals, clinics, health centers) to get in touch with us. We supplemented our sample by recruiting students via personal connections. We purposively sampled [26] medical students from different backgrounds –covering the entire spectrum of Dutch students, international students and students placed in different healthcare settings, to ensure diversity in student characteristics, work environments and study mode (e.g., part-time). Students who showed interest in the interviews were asked to share their final clerkship request, approved by the faculty, and, thereafter, received the information letter. Once students agreed to participate in the study and had signed the consent form, either an online or an in-person appointment was scheduled to conduct the interview. In-person interviews were held at the medical faculty, and participants did not receive an honorarium for their time. We included students who had started their final clinical placement within 12 months before this study started, or had their clinical placement confirmed and were about to start within 9 months of the commencement of this study. We considered this sample size appropriate for our exploratory study, as it provided rich and meaningful information power to capture students’ experiences [27] and to answer the research question.

### Data collection

From January to July 2024, the second author (JGB) and a fellow medical student conducted semi-structured interviews with all participants. This setup was chosen to ensure that interviewees felt comfortable sharing information with a peer they would not otherwise disclose to an educator/faculty member. We speculated that the interviewer role as a medical student would minimize power dynamics. Nonetheless, there were no pre-existing relationships between the interviewer and any of the participants. The interviews were guided by a flexible interview guide *(Additional file)*, allowing the interviewer to follow the interviewees’ lead and probe further into areas they identified as significant. To ensure rigor [28], JGB made field notes after every interview and debriefed with EM to discuss the responses of the interviewees. The debriefings provided additional insights into underexplored topics that seemed relevant to the research question.

The interviews, which lasted 35 to 90 minutes, were audio-recorded and transcribed verbatim by the second author. Transcriptions included both verbal content and notable pauses or hesitations, in line with our interpretive approach to qualitative analysis [29]. Data collection was continued until no new information was obtained from students’ accounts of what factors they considered important when choosing their clinical placements.

### Data analysis

JGB was responsible for conducting the interviews and collecting the data (audio transcripts and descriptive characteristics of students). We used Atlas.ti to manage and analyze the data. AA and JGB coded the transcripts iteratively and constantly compared new data and interpretations with previous assumptions and understandings. This resulted in the following analytical steps:

1. *Familiarization with the data*: Researchers JGB and AA familiarized themselves with the data by reading all the transcripts.
2. *Preliminary open coding of the data:* JGB and AA inductively coded the first three transcripts line by line independently, discussing them in detail, interpreting and identifying the first codes related to understanding what students considered important factors when coordinating their final clinical placements. The two met weekly to compare the codes, a process that allowed additions, subtractions, or adjustments to the codes. A draft codebook was created from the initial codes deemed relevant to answering the research question (Appendix).
3. *Development of coding template:* JGB and AA moved from coding to searching for initial themes and, thereafter, developed a preliminary coding template for the first subset of the data. The template was then discussed with the rest of the research team (EA, MACF).
4. *Application of coding template and revision*: JGB and AA coded ten more transcripts, thereby generating new codes when necessary to capture new meanings, not yet covered by the preliminary codes or template. They then applied the coding template to these transcripts, adapting it when needed.
5. *Final interpretation:* The final template was discussed in team sessions (JGB, AA, EA, MACF) and was adapted as needed by consensus. EA and MACF advised when necessary to resolve uncertainty and reflected on the data from their background. Based on their remarks, we adjusted the template after agreeing that it would better justify the data. JGB went on to code the remaining three interviews, simultaneously adapting the template. Once this was done multiple times with no new codes emerging, the template was assumed to be a valid representation of the data.
6. *Writing the report:* In the final phase of the analysis, we browsed through and selected the most representative examples of the themes and subthemes within our template. We then used those to generate the final extract narrative in relation to the research question.

### Research team and reflexivity

Our research team varied in disciplinary perspectives and included researchers with complementary expertise we judged relevant to the data analysis process. All members have had clerkship experiences that influenced our insights in this research process. An internal medicine clinician and examiner for the clinical clerkship module (EM), a main teacher in the clinical clerkship module (BTtH), a medical student (JGB) who conducted the interviews, and an MD-PhD student in medical education (AA) all worked in the context of the research. Their expertise and experience had logistical advantages and helped the research team understand the participants’ views and terminology. However, being an insider could have led to an assumed understanding of participants and a loss of objectivity [30]. Therefore, we strived to build a research team with various backgrounds and perspectives. We consequently consulted a higher education researcher who worked outside the research context: a full professor in health professions education (HPE) research with a background in internal medicine (MACF) and clinical teaching experience. MACF’s wealth of experience with medical students in training enabled him to bridge the clinical and educational perspectives.

We sought to understand the meaning that participants gave to their (learning) experiences and used those experiences to gain insight into factors influencing students’ clerkship choices. We took an active role in making sense of the data in light of our research aims, reflecting on how our own perspectives shaped the analytic process. Rather than simply summarizing or reporting our data, we interpreted it in relation to our assumptions about students’ decision-making. One team member approached their own decision from a logistical standpoint, selecting an academic hospital placement to align with plans for a scientific internship abroad. Another recalled choosing a clerkship while trying to ascertain whether that particular medical practice aligned with their perceived future aspirations. A third team member, despite being shaped by financial hardship and limited social support, chose to pursue a genuine passion for the field. Our varying perspectives reminded us to approach students’ narratives without imposing assumptions of how decisions *should* be made, and instead to honor the complexity and individuality of these choices.

## Results

Of the invited students, 16 agreed to participate and were all subsequently interviewed. The rest of the students who did not respond to our invitation were not contacted further. We did not notice any connection between participants’ different backgrounds and their narratives. Participants described their final clerkship choice as a dynamic, complex process involving many interacting factors. Characteristics of the participants are shown in Table 1.

**Table 1:** Characteristics of study participants.


ATTRIBUTE	NUMBER OF PARTICIPANTS

*Gender*	

Female	10

Male	6

*Age (range)*	

23–28 years	16

*Nationality*	

Dutch	11

non-Dutch	5

*Clerkship choice (core)*	

Neurology	3

Pediatrics	1

General practice	1

Surgery	2

Internal medicine/ER	3

Social medicine	1

Psychiatry	1

Dermatology	1

Rehabilitation care	2

Rheumatology	1


The participants in our study communicated their experiences in four main themes that we elaborated from the data: ‘emotions involved in making the decision’, ‘between now and next’, ‘the learning environment beyond the clerkship’ and ‘decisions under structural pressures’ (Templates 1, 2, 3, 4). The frequent use of feelings, metaphors, and graphical descriptions suggested that discussing the rationale and the factors behind their choice offered students an opportunity to express themselves openly. As one participant stated when describing his dilemma in choosing the final clerkship: *“It’s like a railroad crossing where many things need to align to lead to something”* (T_57). Participants reported indeed that the experience of choosing their final clerkship was challenging. Students experienced this decision-making task as an exercise that required a lot of information processing. In the following sections, we will elaborate on each theme in detail. We present each theme along with a specific template with respective coded factors.

**Template 1:** Emotions involved in making the decision.


CODES	SUB-CODES

**Stress**	- Stress resulting from uncertainties (and peer influence) - Stress resulting from having to justify/motivate the choice of the final clerkship - Stress from not completing the clerkship on time, staying behind and delaying master thesis

**Happiness**	- Feeling happy with the exposure during earlier completed clerkships - Feeling happy knowing they have experienced a ‘friendly-atmosphere’ department that they can return to

**Reassurance**	- Feeling reassured of becoming a meaningful doctor

**Hope**	- Hoping to feel valued as a young member of the team - Hoping to encounter an enjoyable working atmosphere - Hoping to feel reassured with level of professional competence


**Template 2:** Between now and next.


CODES	SUB-CODES

Conscious individual elements	- Well-informed based on individual exploration - Timeline based on further education ((non)-specialist training) in the same field - Having protected time available

(Influence of) family, peers and friends	- Nurture effect - Romantic relationships - Common medical student ‘chosen’ departments/hospitals

Geographical location	- Residing in the same city - Urban activities of preference (cultural clubs, student associations) - Avoiding housing/accommodation challenges - Commuting (travel time) between home and work - The need to find new housing - Not being separated from pets - Being physically close to big sport events (football clubs)

Work-life balance	- Future family prospects when working in departments with low patient load - Part-time job possibilities in extramural healthcare - Limited emotional workload when working with certain patient groups


**Template 3:** The learning environment beyond the clerkship.


CODES	SUB-CODES

Collegial Influences	- Good collaborative working atmosphere - Easy accessibility Administration when reaching out to the department Safe and friendly/informal atmosphere - Role model(s) Being able to gain trust from colleagues quickly - Little hierarchical relations within the discipline - Reputation of the hospital based on peers’ views

Supervisor(s) Mentorship	- Personal connection with supervisor Autonomy-satisfying supervision style

Future Career Thoughts	- Gaining knowledge and skills during the final clerkship Building relevant clinical (work) experience (bedside rounds, inpatient wards) - Patients (Increased) patient contact desired Patient population groups Preference towards age groups (e.g., pediatrics/geriatrics)

International Students’ Background(s)	- Language of communication in home country


**Template 4:** Decisions under structural pressures.


CODES	SUB-CODES

Educational System Influences	- Administration challenges Regulation on duration of core/short elective clerkship Regulation on continuity between core/short elective clerkship Unresponsiveness of particular departments - Competition between students and clerkship places offered

M1, M2 and M3 Experiences	- Order of completed clerkships and Master thesis - Not having been placed in a popular clerkship during M1/M2 placement

Students’ Assumptions/Obligations	- Assumptions/obligations based on beliefs Assumptions of (medical student) role Assumptions that other specialties are more important than others Assuming having to make a decision because of time pressure The idea of having an obligation toward the faculty when choosing the final clerkship Information gaps (not knowing enough about how they should deal with the administration)


### Emotions involved in making the decision (Template 1)

Students experienced a variety of emotional changes as they chose their final clerkship, described as *“growing up from being students to being student doctors” (T_57)*. Stressful feelings stemmed from students’ uncertainties about rationalizing their preferences, beyond simply finding them enjoyable. What also caused stress was the thought of needing to repeat the final clerkship – if not sufficiently passed or deemed suitable – and hence staying behind, often needing to make up for the failed clerkship and revisiting the decision-making process. Namely, one student mentioned: *“It feels like a race—everyone is making choices, and I fear making the wrong one could set me back* (T_54)*”*. These pressures made the decision feel high-stakes, forcing students to choose purely out of desperation to start their clerkship and avoid contemplating it, and to make sure they would graduate in time with the rest of their cohort. While the order of the clerkship and the master thesis was flexible, and students could also take time off during the final master’s year, this freedom introduced uncertainty.

In other cases, students appeared to delay their graduation, either intentionally or implicitly, as they grappled with indecision about their future career steps. For example, a student with an international background expressed reluctance and distress about returning to their home country as a graduate doctor, which seemed to contribute to the continued postponement of their final clerkship. Accordingly, having to move geographically and not being able to keep their stability in accommodation proved highly anxiety-causing for numerous medical students. One student shared: *“Housing also played a role. Having found a house here was a safe option. I couldn’t have gone looking for accommodation again (T_69)”*.

At the same time, students described how the happiness they felt from past clinical experiences was a strong enough motivational factor for them to choose that very same department for their final clerkship. Feeling happy might have been due to having had the exposure to a certain discipline; and hence had ticked the box of experiencing it, or because students felt they would be gladly welcomed back as seniors: *“I was really happy I had neurology in M2. Working there felt like a warm bath, everyone was friendly and made me feel part of the team—I knew I could go back to experience it again (T_06)”*. Such moments of satisfaction reinforced students’ confidence in their choices and provide a sense of belonging to the team of healthcare professionals.

Students also identified hope as a crucial factor shaping their choice of final clerkship. Many desired a sense of value within their clinical team: *“I hope in that department I could feel like a real team member, not just an observer—where I can contribute and learn (T_08)”*. Likewise, the expected feeling of being valued as a co-worker sparked this eagerness to transition from junior to senior clerks. One student namely, described: *“Once I realized that this choice is just a step toward becoming a competent doctor*, *I started feeling reassured even if I did not know the department I ended up in (T_41)”*. Ultimately, the clerkship choice was not just an academic decision but an emotional journey that shaped students’ identity as future doctors. Students realized that this clerkship would be the longest and final of their training, which led them to contemplate aspects such as where, with whom, and how they would like to work on a daily basis as physicians. This reflection was accompanied by students trying to answer the question, ‘What doctor do I want to become?’. Although most students were unable to concretize their answers, they all elaborated on the physician’s profession—what they believe is right and meaningful—and how they *hoped* to discover whether their interests matched their career goals during the final clerkship.

### Between now and next (Template 2)

Students’ choices regarding their final clerkship were also influenced by current practical considerations rather than purely future aspirations. Numerous students selected their clerkship based on whether certain departments offered greater opportunities for future employment as a junior doctor or for entry into specialty training. Students’ words often reflected how medical careers are perceived as following a linear path—from completing a six-year medical degree (bachelor’s and master’s), to gaining experience as a junior doctor, and subsequently entering specialty training. Choosing a department with strong training prospects was therefore seen as a deliberate step toward progressing along this expected trajectory.

Working as a junior doctor in this sense, would mean gaining clinical experience before committing to a specialty, rather than following a structured specialist training. One student shared: *“I saw this as a chance to get my foot in the door. I knew they take a lot of AIOS (doctors in specialty training) so maybe if I showed my dedication early*, *it could help me secure a spot later, if I would want to specialize (T_69)”*. Similar thinking, however, also included students’ choosing a department that allowed them to have protected time after balancing clinical responsibilities with or without other academic pursuits (e.g., conducting research, teaching at the faculty level). One student mentioned: *“I could not stay in surgery. I needed to make sure I’d have time to unload all information when I go home or time to prepare evening tutor groups (T_57)”*.

External influences from individuals were also pivotal in shaping clerkship choices. Students described how they often accepted clerkship advice from romantic partners and family members who knew them well. In this sense, students relied on their significant others to nurture their trust in which specialty would suit their own wants and preferences. Similarly, students often leaned toward hospitals or departments that were already popular among their peers, so as not to feel alone during clinical duty: *“Most of my friends were choosing internal medicine, so it felt like the natural choice. I knew I wouldn’t be alone (T_55)”*. Geographic considerations also played a key role, with students prioritizing placements in cities where they already lived, had social ties (e.g., cultural associations) or engaged in extracurricular activities like student governing bodies. In that sense, students looked forward to having the same amount of leisure time as in previous years of clinical training and did not want to risk losing it at the cost of their final clerkships. One student articulated: *“I wanted to stay in Groningen and so moving wasn’t an option. This way I could still go to the football stadium every weekend (T_04)”*. Finally, when elaborating on the importance of time stressors, students stressed that the future workload volume was sufficient to draw them to departments in extramural care that offered better working hours. One student shared: *“Gynecology would have been tough with all the unplanned hours so I thought something else [extramural] would be a better option. […] I am also more into talking and having longer conversations and not into fast-paced hospital consultations (T_06).”* Ultimately, students’ clerkship decisions were deeply intertwined with their present circumstances, reflecting a balance between personal, social, and near-future career motivations.

### The learning environment beyond the clerkship (Template 3)

The learning environment within a department was the third theme that mattered to students when choosing their final clerkships. An informal, friendly, collegial atmosphere, ease of technological access (email responsiveness), and the presence of inspiring role models all contributed to a desirable learning environment for students. Students specifically valued a work culture where they could quickly gain trust and integrate into the team: *“ I found the people in the GP practice nicer, more social, and they were more interested in each other. It was different (T_36)”*. Adding to the desirable learning environment was also the anticipation of facing little hierarchical intra- and interprofessional relations within the chosen discipline. For example, one student added: *“I find it very important to be able to ask questions and not to just have to answer them. I found clerkships less enjoyable where people tell me what to do without really explaining anything. I think 80% of how much I like a clerkship depends on the clear guidance and communication from the seniors (T_38)”*.

Low-threshold supervision and mentorship were also key factors in the decision-making process. A strong personal connection with a supervisor, combined with an autonomy-supportive supervision style that allowed for open discussions with clerks, made the department more appealing. A student explained: *“I felt like my supervisor really welcomed my perspective. She gave me responsibilities and guidance when I needed, and we also reflected on our days together (T_41)”*. In the learning environment, students also mentioned specific elements they sought to acquire in their target department. These elements included being taught practical skills (such as surgical suturing and intubation) and being exposed to specific patient populations.

Students felt that the more time they spent practicing professional competencies in specific departments, the better prepared they would be for their work as junior doctors and, as a result, avoid unintentional mistakes due to their lack of experience. Likewise, a student mentioned: *“I knew I wanted to work with children, so choosing pediatrics for my final clerkship felt like the best way to prepare myself for working with babies (T_38)”*.

For international, non-Dutch students, the language of communication in the department was an additional concern, as they sought learning environments where they could communicate more effectively, in the language of their choice and in a safer manner: *“I wanted to be in a place where I could fully engage with both patients and staff without language barriers. In my previous [Dutch-speaking] clerkships I had to think twice before saying anything (T_04)”*. These students described the learning environment of their desired clerkship as a respectful and supportive culture where they felt comfortable taking initiative in patient care and were not dependent on their supervisor’s approval for clinical decision-making or for Dutch language skills.

### Decisions under structural pressures (Template 4)

The fourth theme of factors influencing students’ clerkship choices concerned their decision-making in response to administrative principles or pressure from their duties as medical students. Multiple students faced limited placement availability in popular departments such as surgery and pediatrics, leading them to opt for secondary choices that were previously seen as fallbacks. The urgency to secure a spot also created time pressure constraints, forcing students to make decisions quickly to avoid missing out or further uncertainty. As one student noted, *“During our coach meetings, there was almost one person every two weeks who had planned something [clerkship placement] and the rest of us would stress even more because we knew the places were becoming less (T_53) and knew we had to decide quickly.”*

Other students chose their final clerkship based on the assumption that certain specialties are more important for medical students to experience during their training. For example, students mentioned that if they had not had an M1/M2 rotation in surgery or internal medicine, they felt ‘unexperienced’ and ‘unworthy’ of graduating as doctors. Missing out on one of these departments translated to lacking in clinical knowledge and skills. Consequently, many clerks chose such a department to address their perceived clinical deficit. As one student shared: *“Everyone says you need to have experience at the ER before graduating. I didn’t want to be the one who had zero ER experience (T_69)”*. Likewise, students chose an arbitrary, available clerkship placement to avoid wasting time thinking about their master’s thesis topic. This way, they could complete their final clerkship and use this time to contemplate the planning of their dissertation (and eventual graduation): *“I did not want to just wait. I wanted to continue with the final clerkship so I could use that time to prepare for the thesis without any delays (T_36)”*.

Decisions were also influenced by students’ struggles with a lack of clear guidance on how to navigate the administrative process for applying for the final clerkship. The fact that students did not know which steps to follow or in what order to apply for and reserve their placement led to a tendency to settle on something familiar or previously set up by coordinators or peers. Characteristically, one student said: *“I honestly didn’t even know how to apply for clerkships outside of the region, because there are many steps you need to go through on Scorion [the student portfolio] and so I just responded to an email from the hospital coordinator which seemed easiest to arrange (T_06)”*. Ultimately, many students chose arbitrary clerkships under pressure to justify their choice within a certain deadline set by the faculty (e.g., submitting the necessary application within 9 months of the preliminary starting date).

## Discussion

In this study, we examined the factors that influence final-year medical students’ choice of clinical clerkships. Our findings provide insight into how an evolving interplay of personal, social and academic influences shapes students’ experiences of their clerkship decision-making process. We found that choosing a final clerkship is an emotional and oftentimes stressful process for students, involving thoughtful consideration of the many possibilities for their future careers. We also found that medical students currently undergoing clerkships attach great importance to well-being, peer-suggested influences, social support networks, and finding specialties that match their values. In the rest of the discussion, we comment on the factors that are important to current medical students when choosing their final clinical placement(s) and the clinical contexts in which they expect to work.

Our findings show that medical students simultaneously weigh which future roles in the clinical workplace they would be a good fit for, and what experiences they need to prepare for those roles. Students face important future-oriented decisions that need to be made ‘now’, while contemplating multiple possibilities and opportunities to expand their professional careers [31]. Recognizing this phase of medical training as exploratory, it is vital to understand how young graduates envision becoming resilient healthcare workers [32]. By distancing themselves from competition-driven departments and authoritarian supervisors, students protect their ability to thrive and, therefore, to provide optimal patient care [33]. Similar to our findings, research highlights how healthcare professionals will remain long undecided (and potentially unemployed) before compromising between or combining interests, capacities, values, and available favorable opportunities in clinical practice [34].

Our study also uncovered that future physicians are motivated by wellbeing-friendly specialties characterized by reduced working hours, manageable patient workloads, and a satisfying work-life balance. These findings are further supported by research in similar age groups [35] within and outside hospital settings [36] and across multiple health professions [34]. In this sense, healthcare workers have a stronger desire to uphold principles of limited working conditions and reliable organizational support, and they dare to advocate for their personal well-being [37]. We speculate that this orientation reflects current trends in structural changes within (inter)national healthcare environments and how graduates are responding to these. Specifically, Dutch data indicate that occupational risk factors such as physically demanding work, low autonomy, a high workload, and poor intercollegial communication are the most significant predictors of burnout [38,39,40]. Similar trends have been observed in shifting from intramural to extramural care due to the intensification of administrative responsibilities in hospital settings [41] and increasing time pressures [42–43]. As a result, once students feel their learning environment is professionally rigid, lacks clear communication, or does not support their lifestyle, they feel threatened and begin to reconsider their positionality in clinical care, which can be emotionally draining [9]. Echoing prior work, our findings support the emphasis of lifestyle considerations in physicians’ career trajectories; thereby keeping the medical profession attractive and avoiding larger gaps in healthcare personnel shortages.

Additionally, we found that students preferred to consult individuals they are comfortable talking to, trust, and know well —to help them visualize the kind of work they could thrive in and provide good patient care. This tendency to seek academic and career-related guidance from trusted social and romantic relationships reflects a thoughtful approach to decision-making, one that values familiarity, trust, and personal insight [44–45]. It also reflects this generation’s need for focused career counselling [46]. After all, students thought there was a greater need for career counselling from the university, or at least a scheme outlining the process for finding or applying for a suitable placement. Being aware of students’ preferences in clerkship placements, clinical educators and administrators can inform their guidance, thereby adjusting it to more effectively support students’ learning in the workplace.

Our study has several limitations. First, it was conducted at a single medical school in the Netherlands, and its findings may not generalize to international medical schools where curricular variation and cultural differences are important variables. For example, emergency medicine is not part of our students’ core clinical rotations but is mandatory during the clinical phases at numerous medical schools internationally [47,48]. Medical education programs in other countries—particularly those with extended clinical training phases of 2–3 years followed by a year-long internship—may offer students broader exposure to a wider range of specialties before they choose their final area of focus.

Medical education programs.

Second, the peer status of the interviewer—as a fellow medical student —may have unintentionally shaped the interview dynamics. While this likely encouraged openness and candor, it may also have introduced response bias—participants may have assumed shared understanding or omitted clarifications. In addition, the insider positionality of our research team, all affiliated with the same institution, could have influenced both data interpretation and analysis. We attempted to mitigate these effects through reflexive dialogue and regular debriefing, but acknowledge that complete objectivity is unattainable.

As a next step, we suggest implementing tailored mentorship programs in medical education: by noticing how students grapple, clinical mentors can effectively support them in their efforts to get to know themselves and the professional opportunities they can explore. In addition, we believe future research could examine whether medical students’ clerkship choices influence the medical specialization they ultimately pursue.

### Practical implications

Adapting clinical education for future physicians isn’t about catering to them—it’s about evolution; ensuring that the medical profession remains attractive and sustainable by recognizing the changing expectations, values, and needs of younger physicians. To better support students in making critical, impactful career decisions, faculty should engage in ongoing, individualized mentoring relationships that extend beyond clinical instruction. Medical graduates are navigating uncertainty- trying to make sense of what they know, anticipate unpredictable futures, and chart a course toward roles where they’ll thrive, all while determining the steps they need to take to get there. Importantly, students require mentorship that supports both their personal well-being and professional development.

Clinical mentors should be equipped not only with knowledge of the job market and (non-linear) clinical pathways but also with a genuine understanding of each student’s goals, values, and support needs. This is a call for educators to integrate career counselling into medical education, encouraging open dialogue about the importance of social support networks and the value of students’ habits and preferences for their well-being. Career counsellors should recognize and respect that students are thoughtfully considering the types of support they need to thrive in their professional lives. Rather than dismissing factors such as social support and personal boundaries as irrelevant to the workplace, mentors should engage with these concerns as integral to career decision-making success. When such guidance is absent, students risk entering specialties misaligned with their strengths or values, potentially leading to dissatisfaction, burnout, or attrition. This misalignment can further exacerbate workforce shortages and disrupt continuity of care. Thoughtful, student-centered career advising contributes to a more resilient, well-supported healthcare workforce that is better prepared to meet both personal and systemic demands.

## Additional File

The additional file for this article can be found as follows:

10.5334/pme.1747.s1 Additional file. Interview guide.

## Appendix – Draft codebook

### Emotions/feelings experienced in the process of choosing the final clerkship

✤ Fear
✤ Doubt(s)
✤ Stress
✤ Worries/concerns about future career
✤ Happiness
✤ Hope
✤ Feeling valued/feeling a meaningful doctor

### Interpersonal elements

✤ Development of skills or values
✤ Assumptions/obligations
✤ Preferences based on current working/learning environment
✤ Future career thoughts

### Relational elements

✤ Social influences on the choice of the final clerkship
✤ Atmosphere of the working/learning environment
✤ Assumptions/obligations
✤ Educational system/faculty influences on the choice of the final clerkship
