# Supplementary material for: Caught Between Now and Next: A Qualitative Study into Final-Year Medical Students’ Clerkship Choices
Source: Perspect Med Educ. 2025 Nov 14;14(1):813–25. doi: 10.5334/pme.1747 (PMC12617418; doi:10.5334/pme.1747)
Supplement: Additional file. — Interview guide. [file pme-14-1-1747-s1.pdf]

Additional file (Interview guide)

Interview questions

How did you experience the process of choosing your final clerkship placement?

Influencing factors

Which factors influenced your choice for the final clerkship placement? Why?

a) Which factors outside the hospital influenced your choice of the final clerkship? How?
b) How did your previous experiences influence your choice of the final clerkship?
c) To what extent did the clerkships in M1 and M2 influence your choice of the final clerkship?
d) To what extent did personal circumstances influence your choice?
e) To what extent did the possibilities of acquiring knowledge and developing skills (CanMeds competence development) in a specific specialty play a role in making your choice?
f) Finally, are there any other factors that you haven’t shared that influenced your choice of the final clerkship?
g) In retrospect, what was the most important factor for you that played a role in your choice of the final clerkship?

Personal factors

a) How did you feel during this process of choosing your final clerkship? How stressful of a process was it for you? Why?
b) To what extent did you consider future career prospects when choosing your final clerkship?
c) To what extent did the workload of the discipline/specialty influence your choice of the final clerkship?

Making the choice

Why did you choose this specific core clerkship?

a) In retrospect, would you have preferred to make a different choice and if so, why? On which factors was this choice based and how do these factors play a role in this?

Supervision/Guidance

What was the supervision/guidance you received like in making your choice for the final clerkship placement?

a) Which individuals influenced your choice for the final clerkship and how did they do so?
b) How did you experience the guidance from the faculty in making your choice for the final clerkship?

Do you know other medical students from the university who based their final clerkship choice on other factors?

Do you have any questions?
